# Disability caused by medication-overuse headache can be considerably reduced by detoxification. Results from multinational COMOESTAS study

**DOI:** 10.1186/1129-2377-1-S14-P227

**Published:** 2013-02-21

**Authors:** L Bendtsen, R Jensen, S Munksgaard, C Tassorelli, G Nappi, Z Katsarava, M Lainez, J Leston, R Fadic, R Jensen

**Affiliations:** 1 Danish Headache Centre, Dept. of Neurology, Glostrup Hospital, Faculty of Health Sciences, Universit, Denmark; 2 Danish Headache Center, Department of Neurology, Denmark; 3 Headache Science Centre, Mondino” Foundation and Department of Public Health and Neurosciences, Univ, Italy; 4 Department of Neurology, University of Essen, Essen, Germany; 5 Fundación de la Comunidad Valenciana para la Investigación Biomédica, del Hospital Clínico Universit, Spain; 6 Fundacion para la Lucha contra las Enfermedades Neurologicas de la Infanzia, Buenos Aires, Argentina; 7 Pontificia Universidad Catolica de Chile, Santiago, Chile

## Introduction

Medication overuse headache (MOH) is a common, disabling and costly disease that it is potentially treatable. Several studies have demonstrated significant reductions in headache frequency after detoxification. From the patients perspective, the effect on disability, anxiety and depression is often equally important. This has been less often examined and mainly in single centre studies.

## Objectives

To investigate whether headache-related disability, depression and anxiety can be reduced by detoxification. Methods Patients with MOH were included from 6 centres in South America and Europe. Before and 6 months after detoxification, the degree of disability was measured by The Migraine Disability Assessment (MIDAS) score, while anxiety and depression were measured by the Hospital Anxiety and Depression Scale (HADS).

## Results

A total of 692 patients with MOH were included of which 519 completed the study. Headache days were reduced from 23.6 to 9.8 per month (p<0.001). The MIDAS score was reduced from baseline 59.8 to 25.5 at 6 months after detoxification (p<0.001). HADS depression score was reduced from 6.6 to 4.1, while HADS anxiety score was reduced from 9.3 to 7.1 (both p<0.001).

## Conclusion

Disability, depression and anxiety were considerably reduced in patients with MOH by detoxification. This emphasizes the urgent need for increased awareness about avoiding overuse of headache medications both among the public and professionals and demonstrates that not only headache frequency but also quality of life are remarkably improved by detoxification.

## Conflict of interests

None.

